# On Refining the SZZ Algorithm with Bug Discussion Data

**DOI:** 10.1007/s10664-024-10511-2

**Published:** 2024-07-24

**Authors:** Pooja Rani, Fernando Petrulio, Alberto Bacchelli

**Affiliations:** Department of Informatics, University of Zurich, Zurich, Switzerland

**Keywords:** Software quality, Pull request, Empirical research, Taxonomy, SZZ algorithm, Bug-introducing commits, Mozilla

## Abstract

**Context:**

Researchers testing hypotheses related to factors leading to low-quality software often rely on historical data, specifically on details regarding when defects were introduced into a codebase of interest. The prevailing techniques to determine the introduction of defects revolve around variants of the SZZ algorithm. This algorithm leverages information on the lines modified during a bug-fixing commit and finds when these lines were last modified, thereby identifying bug-introducing commits.

**Objectives:**

Despite several improvements and variants, SZZ struggles with accuracy, especially in cases of unrelated modifications or that touch files not involved in the introduction of the bug in the version control systems (aka *tangled commit* and *ghost commits*).

**Methods:**

Our research investigates whether and how incorporating content retrieved from bug discussions can address these issues by identifying the related and external files and thus improve the efficacy of the SZZ algorithm.

**Results:**

To conduct our investigation, we take advantage of the links manually inserted by Mozilla developers in bug reports to signal which commits inserted bugs. Thus, we prepared the dataset, *RoTEB*, comprised of 12,472 bug reports. We first manually inspect a sample of 369 bug reports related to these bug-fixing or bug-introducing commits and investigate whether the files mentioned in these reports could be useful for SZZ. After we found evidence that the mentioned files are relevant, we augment SZZ with this information, using different strategies, and evaluate the resulting approach against multiple SZZ variations.

**Conclusion:**

We define a taxonomy outlining the rationale behind developers’ references to diverse files in their discussions. We observe that bug discussions often mention files relevant to enhancing the SZZ algorithm’s efficacy. Then, we verify that integrating these file references augments the precision of SZZ in pinpointing bug-introducing commits. Yet, it does not markedly influence recall. These results deepen our comprehension of the usefulness of bug discussions for SZZ. Future work can leverage our dataset and explore other techniques to further address the problem of tangled commits and ghost commits. Data & material: https://zenodo.org/records/11484723.

## Introduction

Software engineering researchers conduct empirical studies to understand the cause and impact of poor software quality Bird et al. (2011); Wehaibi et al. (2016); Ray et al. (2016); Kamei et al. (2012). Particularly insightful in this context is knowing which specific code changes led to the introduction of bugs (henceforth, *bug-introducing* changes).https://zenodo.org/records/11484723 For example, researchers have used this information to understand the effect of ownership Bird et al. (2011) and technical debt Wehaibi et al. (2016) on software quality, to study the relationship between code naturalness and riskiness Ray et al. (2016), and to investigate which changes can introduce bugs Chen and Jiang (2019).

**Fig. 1:**
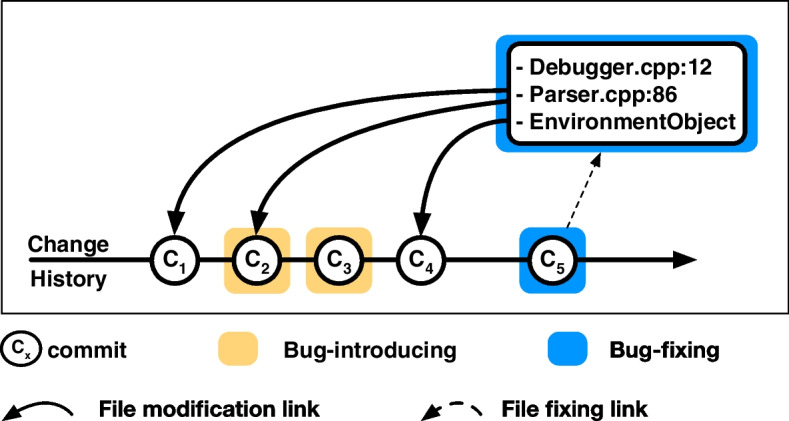
Normal workflow of SZZ to identify *bug-introducing* commit(s) from a *bug-fixing* commit (File names taken from Example: [*Bug:1691941*])

Information regarding which changes introduced bugs must typically be inferred. Methods to do so are generally based on an analysis of the changes that rectify the bug (*bug-fixing* changes), a detail that is more commonly accessible. The SZZ algorithm, proposed by Sliwersky, Zimmermann, and Zeller Śliwerski et al. (2005), stands out as one of the foundational and most widespread methods. This algorithm uses VCS (version control system) data to identify bug-introducing commits from a given bug-fixing commit. As illustrated in Fig. 1, the identification process involves two primary steps. Initially, SZZ pinpoints bug-fixing commits, often by searching commit messages for keywords associated to bugs, like *fix*, *bug*, or the *bug-id*. Subsequently, for each file addressed in these fixing commits, the algorithm focuses on the lines that are changed or deleted. By tracing these lines through the VCS history, SZZ marks the most recent commit that altered them as *bug-introducing* commits.

The SZZ algorithm operates under several assumptions. For example, SZZ posits that all changes in the bug-fixing commit point to bug-introducing commits. However, the existence and frequency of *tangled commits* Herzig et al. (2016), where many unrelated files are altered within a single commit, challenges this assumption’s validity. Furthermore, SZZ presumes that bug-introducing commits are fixed by modifying the same files that caused the bug. Yet, numerous studies have refuted this notion, demonstrating the frequent presence of *ghost commits* Rezk et al. (2021) and inability of SZZ heuristics to handle them. In these instances, the bug-introducing commit does not overlap with any file from the bug-fixing commit Rodríguez-Pérez et al. (2022, 2020); Petrulio et al. (2022). Such discrepancies can arise, for example, when the fix involves modifying dependent, related, or external files. We scoped our study to the files present in the repository that cause *intrinsic bugs* Rodríguez-Pérez et al. (2022).

To address inaccuracies and reduce the noise in SZZ, researchers have proposed SZZ variants, such as AG-SZZ  Kim et al. (2006), L-SZZ  Davies et al. (2014), and enhancements which include additional data sources, such as commit messages Rosa et al. (2021), bug reports, change log messages Wu et al. (2011), and commits referenced in Pull Requests (PRs) Bludau and Pretschner (2022) to better identify relevant bug-fixing and bug-introducing commits.

**Fig. 2:**
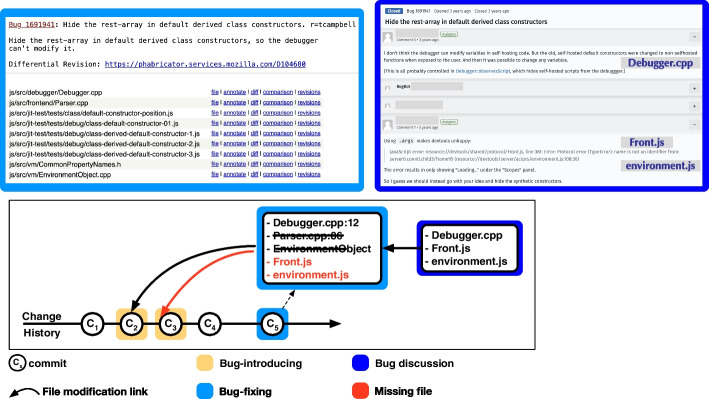
Proposed workflow of SZZ with an hypothetical change history of identifying bug-introducing commit(s) from a bug-fixing commit on a Bug (Example: [*Bug:1691941*])

Despite these enhancements, using bug report discussions for enhancing SZZ ’s capabilities and accuracy is still largely unexplored. Yet, developers can mention related and external files in bug report discussions Mills et al. (2020) Fig. 2 shows an example where two developers discuss relevant files to bug fixes, *e*.*g*., Debugger.cpp, Front.js in [*Bug:1691941*]. It seems reasonable to hypothesize that leveraging these references could enhance the performance of SZZ in identifying true bug-introducing commits. For example, SZZ identifies the modified files in bug-fixing commits (in a hypothetical change history) and traces the recent edits to reach bug-introducing commits as shown in Fig. 2. Among many modified files in bug-fixing commit (C_5_), we can keep the relevant file Debugger.cpp as developer discussed it in the bug report, and we can remove the files that are not discussed, *e*.*g*., Parser.cpp, thus addressing the problem of Tangled commit C_5_. Additionally, we can consider other discussed files such as Front.js, environment.js as these can be related files that might not be modified in the bug-fixing commit but can help reach the bug-introducing commit C_3_. We challenge this hypothesis by investigating whether and to what extent the files mentioned in bug discussions can enhance the recall and precision of the SZZ algorithm in correctly identifying bug-introducing commits.

To conduct our research, we require a dataset where bug-introducing commits are accurately linked to their corresponding bug-fixing commits, and the fixes are initiated and discussed in bug reports. Although most established software projects typically link bug-fixing commits to bug reports, as previously mentioned, the specific origin of a bug is rarely documented. However, Mozilla represents a notable exception. Since 2019, Mozilla developers have been manually identifying and documenting the specific issue reports (which may be regard bugs, but also the implementation of new features and improvements) responsible for the bugs they address, thus connecting bug-fixing commits directly to the bug-introducing ones. This practice allows us to create a dataset that have the characteristics we need for our investigation. Furthermore, Mozilla’s ecosystem offers several advantageous features for a detailed case study. It uses Bugzilla, an openly accessible issue-tracking system, and BugBug Castelluccio (2021), a tool designed to extract essential metadata about issue reports. Additionally, Mozilla employs a broad range of programming languages and develop project of various sizes and domains. Therefore, for our study we have chosen Mozilla as our subject and have compiled a dataset of 12,472 bug reports from its repositories. This selection allows us to explore a comprehensive set of data points, enhancing the relevance and applicability of our findings. In the next step, we conducted a manual analysis of a statistically significant sample of 369 bug discussions from Mozilla. Our goal was to understand the reasons why source code files are mentioned within these discussions and to assess their utility in refining SZZ algorithms. Our findings confirmed the potential in using this additional information to enhance SZZ. We also developed several heuristics to pinpoint files involved in either bug-introducing or bug-fixing commits. These file references in bug discussions allow us to leverage file references to address specific issues for SZZ, namely tangled commits (by excluding non-relevant file references from bug-fixing commits) and ghost commits (by incorporating additional relevant references into bug-fixing commits), as shown in Fig. 2 via an example.

Our results show that including the files mentioned in the bug discussion enhances the effectiveness of SZZ in identifying true bug-introducing commits. However, we found the approach ineffective in handling the problem of ghost commits.

The main contributions of our study include:

- A taxonomy of the reasons why developers mention files in bug reports, which are included in bug-fixing commits, bug-introducing commits, both, or neither them, derived from a qualitative analysis of 369 bug report discussions;
- a novel variant of SZZ, FI-SZZ, that infers relevant files from the bug discussion and uses them to improve the accuracy of identifying bug-introducing commits;
- an empirical evaluation of FI-SZZ showing its effectiveness in handling the problem of tangled commits;
- An extended version of the dataset proposed by Petrulio et al. (2022), named RoTEB (a Repository of Tangled and ghost commits) updated with more recent bugs for a total of 12,472 bugs Anonymous (2023).

## Background and Related Work

We introduce SZZ, its implementation details, heuristics, and limitations, particularly in the context of a multi-commit development model.

### The SZZ Algorithm

In 2005, Śliwerski, Zimmermann, and Zeller, introduced an algorithm, now known as SZZ, to identify commits introducing a bug in a software system Śliwerski et al. (2005). The base version of this algorithm (henceforth, B-SZZ) operates in two stages. Initially, from a pool of all commits, the algorithm locates the commits that rectify a bug (designated as *bug-fixing* commit) employing various heuristics. These heuristics might involve scanning commit messages for terms like *fix* and *bug* or identifiers such as bug-id or a bug’s title. These commits are then associated with a bug from an issue tracking system (ITS). Presently, most ITS platforms, including Bugzilla and GitHub, maintain links between a bug report and their corresponding fixing commits. As such, in contemporary software projects, this link is readily accessible, rendering the initial step superfluous.

The second phase of B-SZZ employs a *diff* tool on the bug-fixing commits (determined in the initial phase) to pinpoint the modified line regions, or hunks, within these commits. This tool outputs a list of hunks, highlighting the discrepancies between a file pre- and post-modification. Subsequently, B-SZZ invokes *annotate* functions, such as git blame, on these altered lines to discern the commits where these lines were most recently edited. These commits are marked as bug-introducing. Given that a single commit can encompass multiple modifications or hunks, which may have been adjusted in diverse preceding commits, multiple bug-introducing candidate commits can emerge, as illustrated in the workflow of SZZ ’s second phase Fig. 1. For a specified bug-fixing commit $C_5$, SZZ recognizes $C_1$, $C_2$, and $C_4$ as bug-introducing commits, given that the files amended in commit $C_5$ were most recently altered in these commits. Here, commits $C_2$ and $C_3$ are genuine bug-introducing commits, whereas $C_1$ and $C_4$ are false positives.

While B-SZZ sets the groundwork for detecting bug-introducing commits, it comes with inherent limitations. For instance, it can yield noisy bug-introducing commits because of tangled commits, or it can overlook bug-introducing commits due to ghost commits Herzig et al. (2016); Rodríguez-Pérez et al. (2022).

### SZZ variants

To improve on the accuracy of the B-SZZ algorithm, the scientific community has worked on refining its approach. We describe some of the most popular variants:

1. **AG-SZZ** Kim et al. (2006): One of the earlier attempts to augment the original algorithm was by Kim et al. (2006), who devised *AG-SZZ*. This variant sidelines modifications that pertain to comments or formatting within commits. Moreover, it discerns alterations in method names and uses annotation graphs to filter style changes.
2. **L-SZZ, R-SZZ** Davies et al. (2014),**MA-SZZ** Da Costa et al. (2016): Drawing inspiration from the principles of *AG-SZZ*, Davies et al. (2014) crafted *L-SZZ* and *R-SZZ*. These strategies filter the outcomes of *AG-SZZ* and establish a one-to-one correlation between bug commits, meaning each *bug-fixing* commit corresponds to a singular *bug-introducing* commit. Specifically, while *L-SZZ* prioritizes the commit with the maximum lines of code alterations amongst *bugin* candidates, *R-SZZ* opts for the most recent commit from the candidate pool. Similarly, another variant named ${\textsc {MA-SZZ}}$ filtered the meta-changes of *AG-SZZ* that are related to merge operation, *e*.*g*., branch changes, merge changes, property changes.
3. **Language-Specific Variations**: A number of *SZZ* derivatives, such as ${\textsc {MA-SZZ}}$ and, *RA-SZZ* Neto et al. (2019), *Open-SZZ* Lenarduzzi et al. (2020) embed heuristics tailored for specific programming languages for improving the base accuracy. *RA-SZZ* filters the refactoring operations (that do not change the program behaviour). *Open-SZZ* is implemented on the basis of *B-SZZ*.
4. **PyDriller**: A practical embodiment of these refined principles is the *PyDriller* tool, conceptualized by Spadini et al. (2018). Grounded in the tenets of *AG-SZZ*, this tool harnesses the git-hyper-blame command to overlook meta changes Da Costa et al. (2016) and refactoring modifications Neto et al. (2018). However, one of its limitations is the need for manual intervention to define the changes to be disregarded within a specific file.

### Challenging Scenarios for SZZ

**Fig. 3:**
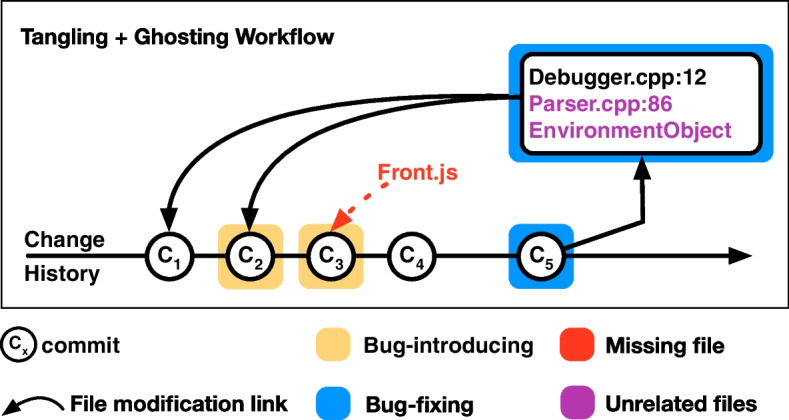
Current workflow of SZZ with Tangled commit ($C_5$) and Ghost commit ($C_3$) for the bug-introducing commit(s) from a bug-fixing commit

Various empirical studies  Herzig et al. (2016); Mills et al. (2020); Herbold et al. (2022a); Fan et al. (2019); Rodríguez-Pérez et al. (2020); Rezk et al. (2021) reveal that the accuracy of SZZ diminishes notably in two specific scenarios, *i*.*e*., with *tangled commits* and *ghost commits*. We depict these two scenarios in Fig. 3 and describe them in further detail.

#### Tangled Commits

A commit is considered *tangled* when it addresses multiple objectives or functions, such as implementing new features, fixing bugs, or refactoring Kawrykow and Robillard (2011). Figure 3 displays the tangled commit $C_5$ where numerous unrelated files might be adjusted. This misleads SZZ to associate this bug-fixing commit with commit $C_1$, even if it is not a bug-introducing commit. Indeed, a number of prior studies have highlighted that tangled commits pose challenges for data analysis and classification algorithms, notably in identifying bug-fixing and bug-introducing commits Herzig et al. (2016); Fan et al. (2019); Mills et al. (2020); Herbold et al. (2022a). Mills et al. (2020) conducted a manual assessment of 2,311 bug-fixing changes spanning 15 projects, revealing that half of these commits were tangled. Herbold et al. (2022a) examined 2,238 bugs across 28 projects to measure the prevalence of tangled commits: they found that only 38% of the modified lines within bug-fixing commits were needed to fix a bug.

As identifying automatically whether a commit, or a set of commits (PR), addresses multiple tasks (*i*.*e*., is tangled) is challenging, we assume PR to be tangled. As SZZ requires at least one file to be shared between bug-fixing and bug-introducing commits to trace bug-introducing commits, we check whether there is at least one common file modified in both bug-introducing and bug-fixing commits. In such a case, we target detangling commits for the bugs that share a file mentioned in bug discussion and modified in bug-fixing commits, as we can keep the relevant files that are discussed in bug reports and remove the noisy files from bug-fixing commits.

#### Ghost Commits

Recent studies have shown that bugs can have different origins Rodríguez-Pérez et al. (2018), which means that some bugs originate in the changes recorded in VCS (project repository) while other bugs are due to the external changes that do not correspond to any alteration within the VCS, *e*.*g*., changes in software dependencies like APIs Rodríguez-Pérez et al. (2020, 2022). A bug is termed *extrinsic* when it arises from changes that are not visible in the VCS and *intrinsic* when the changes are present in the VCS Rodríguez-Pérez et al. (2022, 2020); Da Costa et al. (2016). A case in point from the Mozilla project is bug 1666140,^1^ which pertains to updating the BigInts package of the WebAssembly Javascript Interface—an external package not in the repository. These are known as extrinsic bugs. As the files causing or fixing extrinsic bugs lie outside of the repository (VCS), their bug-fixing or bug-introducing commits do not exist in VCS. If the changes causing the bugs are recorded in VCS, they are known as intrinsic bugs. For some intrinsic bugs, the bug-fixing commits do not modify the files relevant to the bug, and in such cases, the heuristics of SZZ can not trace bug-introducing commits—such bugs are defined as *ghost commits* Rezk et al. (2021). Occasionally, ghost commits manifest between interrelated components of the same software Da Costa et al. (2016); Rezk et al. (2021); Rodríguez-Pérez et al. (2022). Essentially, such a situation arises when the bug-fixing commit solely adds (*e*.*g*., a novel file added to the project) or bug-introducing commit solely deletes lines, or when the files adjusted in the bug-introducing commit differ from those in the bug-fixing commit. In these scenarios, B-SZZ fails to trace the modifications. Ghost commits are predominant in commit-set environments like GitHub and Mercurial. Notably, they account for 45% of bugs in Mozilla Petrulio et al. (2022) and stand as the primary reason for bug-introducing misidentification by SZZ  Herbold et al. (2022a); Rezk et al. (2021).

In our study, we address the challenge of identifying intrinsic ghost bug-introducing commits—instances where bugs are introduced by changes to files in the repository, yet these files are unmodified in the bug-fixing commit, thus rendering the bug-introducing commit untraceable for SZZ. For instance, in Fig. 3, Front.js is not modified in the bug-fixing commit ($C_5$) but is available in VCS and was modified in commit $C_3$—thus $C_3$ is defined as a ghost commit. We investigate whether incorporating files mentioned in bug discussions into the list of potentially relevant files for the bug-fixing commits can address the ghost commits issue and allow SZZ to identify the original bug-introducing commit(s).

### Augmenting SZZ with Additional Information

Bludau and Pretschner (2022) demonstrated the potential of supplemental non-VCS information to enhance SZZ. They incorporated PR messages to associate bug-fixing commits with bugs listed on the issue tracker, resulting in an approximately 18% increase in the overall identification of bug-fixing commits. Although such advancements hold promise in addressing certain ghost commits, they do not address ghost commits in which the bug-fixing and bug-introducing commit do not share any common file between them.

Within issue trackers, developers engage in discussions to comprehend and fix bugs Mills et al. (2020). Earlier research Kochhar et al. (2014); Wang et al. (2015) suggests that bug report discussions often directly reference specific code locations, such as code snippets, files, or class names, providing insights into the potential bug’s location. It is plausible that information extracted from bug report discussions could alleviate issues associated with *tangled commits* (for instance, entities both highlighted in the bug report and addressed by the bug-fixing might likely be initially faulty, rather than components of a separate task) and *ghost commits* (*e*.*g*., entities mentioned in the bug report but untouched in the bug-fixing could pertain to bug-introducing commits which modify those entities. We show an example in Fig. 3. However, the feasibility of supplementing SZZ with this data to mitigate its shortcomings and boost its accuracy remains unexplored. In this study, we set to investigate whether and how bug discussions can improve SZZ performance by adding files discussed in bug reports to the list of relevant files to address the problem of ghost commits and by removing noisy files that are not discussed to address the problem of tangled commits.

## Study Design

### Research Questions

Developers discuss bugs on issue trackers to understand and fix them Mills et al. (2020). Empirical evidence shows that developers often mention files that may have caused the bug or those that can assist in its resolution Kochhar et al. (2014); Wang et al. (2015). Our initial goal to set the basis for our study is to comprehend the reason why source code files are mentioned in bug reports. This analysis expands our understanding of (i) how developers utilize Issue Tracking Systems (ITS) to address and discuss bugs and (ii) the different reasons developers mention files in bug reports. Access to this knowledge can inform the creation of tools supporting various software engineering tasks, such as bug localization, code review, and code documentation. In the context of this study, we examine its potential relevance for the SZZ algorithm. Thus, our first question is:

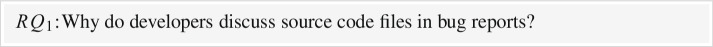



Developers often mention files in bug reports to aid other developers in understanding or fixing the bugs. These mentioned files might be a part of the files modified in bug-introducing commit(s) or bug-fixing commit(s), or not. Recognizing the files that truly belong to bug-introducing and bug-fixing commits can bolster the efficiency of the SZZ algorithm. This is because the SZZ algorithm needs at least one file shared between the bug-introducing or bug-fixing commit(s) to establish a connection. By conducting this analysis, we aim to (i) deepen our comprehension of the frequency with which the files referenced in bug discussions are included in bug-introducing or bug-fixing commits and (ii) identify file references that may enhance the accuracy of the SZZ algorithm in linking such commits. Hence, we ask:

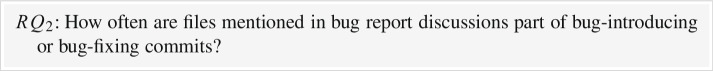



Tangled commits encompass changes that do not relate to the bug fix, such as evolvability changes or other functional modifications. By focusing solely on pertinent files, we can diminish the interference from irrelevant files in SZZ, thus enhancing its precision in pinpointing bug-introducing commits. Our investigation centers on the potential use of files mentioned in bug report discussions for this purpose. Therefore, we ask:

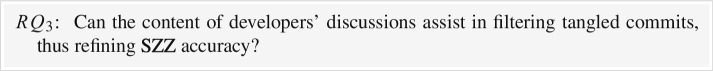



Ghost commits frequently occur in practice Petrulio et al. (2022), but SZZ can not link them to bug-introducing commits using its current heuristics. Consequently, sources of information other than the versioning system become essential. In this research question, we explore the potential of using file references (references to source code files in bug report discussions) to associate files external to the bug-fixing commit with bugs. Should the file be pertinent to the bug, it could direct us to the appropriate bug-introducing change. Hence, we ask:

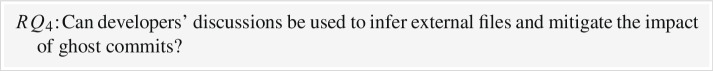



### Data Collection

#### Mozilla Ecosystem

Mozilla is an open-source software community that develops and maintains various software products, such as the Firefox browser and Thunderbird email client.^2^ We sourced bug discussions from the Mozilla ecosystem for various reasons.^3^ First, unlike previous studies that use SZZ to label the bugs Krishna et al. (2015); Koru and Liu (2005); Turhan et al. (2009), Mozilla developers since 2019 started manually referencing the other bugs (with Bug ID) in Bugzilla that have caused this bug or the bugs caused by this bug. It is indicated in Bugzilla using the field *Regressed by* and *Regression* for bug-introducing and bug-fixing respectively. Thus providing the reliable ground truth for identifying and verifying bug-introducing and bug-fixing commits with SZZ.^4^ Second, Mozilla employs an open-source issue tracking system, known as Bugzilla as its primary issue tracker. This platform presents bug discussions, with all remarks pertaining to a specific bug displayed in threaded comments as shown in Fig. 2. Third, the codebase of Mozilla exhibits a wide diversity in terms of domains, project size, and programming languages as shown in Table 1. The table shows the programming languages, the count of files, the number of empty lines (listed under *Blanks*), the quantity of lines with code comments (in *Comments*), and the lines of code (*LOC*) for each language. Lastly, It also provides the BugBug tool Castelluccio (2021), which facilitates exploration of the codebase, its history, and the comprehensive inspection of all bugs discussed upon in Bugzilla. We collect every bug discussion as well as the associated bug-introducing and bug-fixing commits (links created by Mozilla developers), following the strategy by Petrulio et al. (2022) .

**Table 1:** Languages involved in the Mozilla codebase

Language	Files	Blanks	Comments	LOC
**JavaScript**	72,870	1,199,781	1,753,236	5,540,827
**C++**	11,772	801,098	669,043	4,476,606
**HTML**	90,776	463,590	105,185	4,118,159
**C/C++ Header**	16,564	519,357	956,346	2,475,718
**Rust**	8,365	246,505	442,208	2,384,387
**C**	3,998	321,980	502,674	2,158,164
**JSON**	2,245	883	0	1,190,423
**Python**	6,746	222,750	260,302	872,281
**XML**	2,813	7,005	2,973	453,026
**Assembly**	561	35,477	35,924	294,756
**INI**	12,582	73,130	175	231,725
**XHTML**	3,562	23,033	8,097	189,678
**Java**	854	24,503	62,588	156,493
**Other**	11,893	745,785	178,693	1,227,722
**Total**	251,601	4,174,520	4,977,444	25,769,965

#### Bug Report Collection

From Bugzilla, we collected bug reports following various criteria. Our criteria encompassed all bugs from 2019 (since the practice to keep track of bug-introducing and bug-fixing commits in Bugzilla started) marked as closed, resolved, and have the field *Regressed by* to denote the bug-introducing commits, yielding a dataset of 12,472 bugs, each accompanied by its respective discussion. For every bug, we obtained all associated comments from the discussion and extracted any file references contained within. In the extraction process, we preprocessed the comment text, discarding words that neither represented a file nor a file path. To accurately identify file paths, we carried out a preliminary study. Within this study, two authors evaluated comments related to 25 bugs, devising various heuristics to pinpoint the file path. Informed by the findings of this initial exploration, we excluded all file references directed at Mozilla wikis, as bots automatically add these references to the bug discussions. We then assembled the file extensions found within the remaining comments. Due to time and resource limits, we only targeted extensions associated with a minimum of 500 files. As a result, we identified the 29 most prevalent file extensions used in Mozilla’s systems, as detailed in Table 1. Our analysis subsequently revolved around checking whether a comment in a discussion contained any of these identified extensions. The specific implementation details for each research question can be accessed at the provided link.^5^

#### PR Collection

Various studies have shown the increasing trend of using multi-commit development model (e.g, PRs in GitHub) Kalliamvakou et al. (2014); Gousios et al. (2016), as it enables developers to bundle changes together that are spread across multiple commits, *e*.*g*., set of commits to fix a bug. In Mozilla, these sets of commits (bundled commits) are referred to as commit-sets rather than the PRs. For simplicity reasons, we referred to the commit-sets of bug-fixing and bug-introducing commits as bug-fixing PR and bug-introducing PR. Based on the methodology of our previous work Petrulio et al. (2022), we first mined the bugs of Mozilla’s codebase, which contains the links between its bug-introducing and bug-fixing commit-sets via the fields *Regression* and *Regressed By*. Then, we mapped the commit-sets to the individual bug-introducing and bug-fixing commit levels as required by SZZ.

**Table 2:** Description of bugs having the fields *Regressed By* and *Regression* in Bugzilla to extract bug-introducing and bug-fixing PRs (commit-sets) and commits

Cases of bugs with various fields	Bugs	Bug-fixing		Bug-introducing	
		PRs	Commits	PRs	Commits
All bugs	12,472	10,089	14,486	11,879	74,948
Bugs having *Regressed By*	11,879	10,021	14,356	11,879	74,948
Bugs having *Regressed By* and *Regression*	10,021	10,021	14,356	10,021	63,956

Specifically, we used the BugBug tool Castelluccio (2021) to collect the commits with their metadata and all bugs published on Bugzilla. Each commit contains the identifier of the bug it fixes (denoted by the field *Regression* in Bugzilla), and all the commits that fix the same bug represent a bug-fixing commit-sets or PR. To run SZZ on a commit-set, we executed it on each commit belonging to the same commit-set and merged the results later on.

We show the details of 12,472 bugs with the fields of our interest available in Bugzilla, *e*.*g*., bugs have the *Regressed By* field (bug-introducing commit-set), have the *Regression* field (bug-fixing commit-set), or both available as shown in Table 2. Since many bug-fixing commits in our dataset introduces a bug as well, such PRs are known as regression PRs, *e*.*g*., [*Bug:1362919*]. We found 887 such regression PRs (2,110 commits) in our dataset. With BugBug, we also extracted the list of modified files in bug-fixing commits following the methodology by Petrulio et al. (2022). SZZ uses these files to link bug-fixing to bug-introducing commits as it analyzes the changes made in these files and traces the commits where recent edits were performed on the files. Since one bug-fixing commit can contain multiple types of changes, it can lead to multiple bug-introducing commits. Therefore, the relation between *bug-fixing* and *bug-introducing* PRs in our dataset is 1:N.

### Methodology

**Fig. 4:**
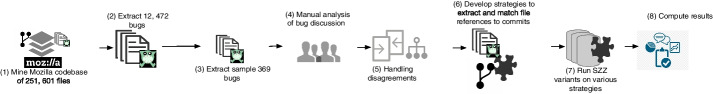
Study Design to perform manual analysis and investigate SZZ on the usefulness of file references to identify bug-introducing commits

We aim to first understand the reasoning behind the file being mentioned in the bug report discussion and then investigate if these mentioned files can help address the challenges of tangled commits and ghost commits as shown in Fig. 4. To this end, we begin by gathering a dataset of bug reports as shown in steps (1) and (2) in Fig. 4. We then formulate various heuristics and strategies to automatically extract discussed or referenced files from the reports to leverage them later on to answer various RQs. Following the heuristics, we analyze a subset of bug reports (*i*.*e*., 369) to first validate our file extraction methods. Then, we manually assess these bugs’ discussions to grasp the reasoning behind the file references in relation to the bug, addressing $RQ_1$, as illustrated in Fig. 4. In the final stage, SZZ adds these file references to the list of relevant files (files modified in bug-fixing commits) to trace bug-introducing commits, aiming to lessen the influence of tangled commits and ghost commits. Specifically, we use file references to investigate whether they can be used to eliminate superfluous files from bug-fixing commits ($RQ_3$) and to reduce the impact of ghost commits ($RQ_4$).

#### $RQ_1$: Rationales behind File Discussion

This research question seeks to extract file references from bug discussion and explore the common reasons why developers reference files in bug report discussions using thematic analysis Braun and Clarke (2006); Coleman and O’Connor (2007).

For every bug in the bug dataset (12,472 bugs), we obtained all associated comments from the bug discussion and extracted any file references it contains. In the extraction process, we preprocessed the comment text to identify file paths, discarding words that neither represented a file nor a file path. To accurately identify file paths, we carried out a preliminary analysis. Within this analysis, two authors evaluated comments related to 25 bugs, devising initial heuristics to pinpoint the file path. Informed by the findings of this initial exploration, we excluded all file references directed at Mozilla wikis, as bots automatically add these references to the bug discussions. We then assembled the file extensions found within the remaining comments. Due to time and resource limits, we only targeted extensions associated with a minimum of 500 files. As a result, we identified the 29 most prevalent file extensions used in Mozilla’s systems, as detailed in Table 1. Our analysis subsequently revolved around checking whether a comment in a discussion contained any of these identified extensions. As a result, we collected 9,281 bug reports where relevant files were discussed. The specific implementation details for finding file paths of various languages can be accessed at the provided link.^6^ After the preliminary analysis, we conducted additional to verify the heuristics and further analyze which of those files are referenced in the discussions and why.

We determined the minimum size of statistically significant samples of bug reports from 9,281 bug reports using the confidence level of 95% and the margin of error to 5% as used in previous studies Rani et al. (2021) using the same formula Triola et al. (2006). As a result, we got a sample size set of 369 bugs.

We sampled the bugs using the random sampling without replacement. Thus, for each bug, we collected its ID, link to the bug discussion in Bugzilla, and the referenced files in the discussion.

The preliminary analysis of 25 samples helped us formulate the initial taxonomy of rationales. Two authors of this paper independently analyzed these sample discussions and described their rationales. Then the authors discussed together their taxonomy to get an initial draft. We further validated and extended this taxonomy with a manual analysis of the statistically significant samples, *i*.*e*., 369 bugs, following a three-phase method. In the first phase, two authors independently examined all the bug discussions and identified the rationale for mentioned files. There can be multiple files mentioned in each bug that can be discussed in different contexts, *e*.*g*., one file can be discussed to explain bug origin while another file can help reproduce the bug. Similarly, one file can also be discussed in different contexts, *e*.*g*., file is mentioned to describe the bug and to solve the bug. Thus, there can be multiple rationales for one bug discussion. Whenever the existing taxonomy of rationale lacks that particular context or is unclear for the author, the author add a new rationale for the bug. For instance, in [*Bug:1691941*] one evaluator identifies that the file is discussed to describe the bug and a potential solution. The other evaluator, in its analysis, also identified that some files are mentioned in the error log. Thus, we independently get two categories for the bug. In the second phase, for each bug the evaluators matched the evaluations of rationales and marked their level of agreement and disagreement cases (with the label *agree*, *partial*, and *disagree*). For example, in the case of [*Bug:1691941*], we found the evaluators agreed partially as the second evaluator found an extra category. The authors agreed wholly on 73% of the cases, partially agreed on 21%, and disagreed on 6% of the cases. The partial agreements were mainly due to missing additional rationales or naming mismatches. The disagreement cases were often due to different formats of reporting files in *System dumps*, *bug description*, and others. Thus, to measure Cohen’s kappa metric, we considered the partial agreements as agreements as both evaluators agreed on the existing rationales and found the value of Cohen’s k metric: 0.859, which is considered an almost perfect agreement between the evaluators. In the third step, the evaluators mutually discussed the disagreement cases and settled on the final list of rationales for each bug. The replication package provides the detailed classified for all bugs Anonymous (2023).

#### $RQ_2$: Linking Referenced Files to Mozilla Ecosystem Files

##### Referenced File Collection

As we want to investigate the usefulness of file references for $RQ_3$ and $RQ_4$, we first extracted the referenced files from the bug reports following the steps described in $RQ_1$. From 12,472 bugs, we found that in 74.41% of the bug reports (9,281 bugs), developers mentioned at least one file. In the next step, we ensure that these files actually exist in the Mozilla codebase. To do so, we mined all modifications performed in Mozilla from its creation until the current version: for each filename, we saved all the file paths related to that filename (*e*.*g*., *file.png*: [--https://www.a/path/to/file.png; --https://www.b/path/to/file.png]). Then, we used this map to parse a file mentioned in a bug discussion into a file that existed in Mozilla repositories’ history. To identify the existence of the mentioned file in the Mozilla system, we applied two strategies:

- *Bug-fixing parsing (FP)*: for each bug, we first tried to match every file mentioned in the bug discussion to one of the files modified in bug-fixing commits, as this ensures that the files involved in bug-fixing commit-sets exist in Mozilla. For the files that are not part of bug-fixing commits, we used the *Relaxed parsing (RP)* strategy.
- *Relaxed parsing (RP)*: for each file mentioned by developers in bug discussion, we select the file path from the Mozilla system with the highest similarity score (described later) if the similarity score between these files paths is greater than 0.5. The threshold is selected based on the manual analysis process and an iterative process to ensure that similar file paths are not discarded. We observed that many files mentioned in the bug discussions contain the local path of the user (running the application or test case); To ensure that such paths are not discarded, we kept the similarity threshold at 0.5 Ramsauer et al. (2019). We also tested with a more strict parsing strategy to see how much the developer mentions the full file path, *i*.*e*., similarity threshold at 0.8; however, we found that it did not include any mentioned file in bug-fixing commits for SZZ input.

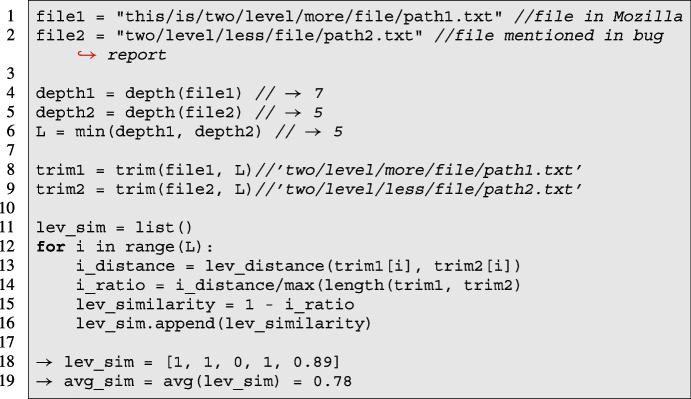


Similar to previous works Ramsauer et al. (2019); Wang et al. (2012); Young et al. (2021), we compared two file paths using Levenshtein distance-based similarity Navarro (2001) as shown in Listing 1. Given two file paths, we defined the maximum similarity length as *L* – the minimum number of common folders in both file paths. The similarity is represented as the mean of Levenshtein similarity between the latest *L* folders in the same position (or level) of the two file paths. We applied the Levenshtein similarity on each folder level to reduce the effect of edit distance across folders of different nesting levels. For example, Listing 1 shows the process of matching two files (file1 and file2) (one file is mentioned in bug discussion, and another is either modified in bug-fixing commits or present in version control history) and computing their Levenshtein similarity.

Once we devise the strategies to match the files mentioned in the bug discussion to the files in the Mozilla system, we check if the mentioned files are part of bug-introducing and bug-fixing commits to answer RQ2 on how often the files mentioned in bug report discussions are modified in those bug commits.

A file mentioned in the bug discussion can be part of a bug-introducing or bug-fixing commit(s), both, or none; we use the following definitions for various cases:

- *FFC:* a *f*iles in the bug discussion are only part of the bug-fixing *c*ommit-set, also referred to as PR
- *FBC:* the *f*iles in the bug discussion are only part of the bug-introducing PR
- *FFBC:* the *f*iles in the bug discussion are part of both (bug-introducing and bug-fixing PR)
- *FNC:* the *f*iles in the bug discussion is not part of either of them

Identifying various cases of whether the files discussed in bug reports are indeed part of bug-fixing or bug-introducing PRs, can help test our hypothesis on the relevance of these file references for SZZ. For example, when the file references discussed in bug reports are modified in bug-fixing commits (FFC, or FFBC), we can use them to remove noisy files from bug-fixing. Similarly, in the case of FBC or FFBC, we can add extra file references to bug-fixing to trace bug-introducing commits. We identified the case information (*e*.*g*., FFC, FBC) by matching the mentioned files in the bug discussion with the files modified in bug-introducing or bug-fixing commits using the FP and RP strategies (as described above). Since 2019, the project’s developers of Mozilla started practices to keep track of the links between the bugs that introduce other bugs in Bugzilla. Each bug contains the field *Regressed by*^7^ to denote that this bug has been introduced by the listed bugs or to denote *bug-introducing* commits and the field *Regression* to denote the *bug-fixing* commit directly by the developers. These fields are labeled by developers in contrast to previous work that uses SZZ to label the bugs Krishna et al. (2015); Koru and Liu (2005); Turhan et al. (2009). This way, we can get a reliable ground truth of such links. We described concerns in Section 2 around SZZ due to its inaccurate heuristics and false positives.

Once we developed the strategies to extract the file references from bug discussion (in RQ1) and match them to the files in Mozilla (RQ2), we applied each strategy independently or in combination on the RoTEB dataset to check the effectiveness of SZZ for

the case of tangled commit ($RQ_3$) and ghost commits ($RQ_4$), as shown in Table 3.

### $RQ_3$ and $RQ_4$: Tangled Commits and Ghost Commits

These RQs focus on using the files mentioned in the bug discussion to investigate whether they could help address the problem of tangled commits and ghost commits in SZZ.

To detangle the commit(s), we should remove files from a bug-fixing commit so that SZZ trace only the modification of files relevant to identify bug-introducing commits; therefore, we leverage the bug discussion files and match them to the Mozilla codebase files using the DFP (Detangle with FP) and DRP (Detangle with RP) strategies, as described in Table 3.

**Table 3:** The file parsing strategies (FP and RP) (devised in RQ2) applied to remove or add files for tangling commits (RQ3) and ghosting commits (RQ4)

Strategy	Strategy Expansion	Description
DFP	Detangle with FP	Remove the noisy files from the bug-fixing commit. The files mentioned in the bug discussion are matched in the Mozilla repository using the FP strategy and the files that do not match are identified as noisy files
DRP	Detangle with RP	Remove the noisy files from the bug-fixing commit. The mentioned files are matched in the Mozilla repository using the RP strategy and the files that do not match are identified as noisy files
URP	Unghost with RP	Add extra files in the bug-fixing commit that are mentioned in the bug discussion and are matched in the Mozilla repository using the RP strategy
DFURP	Detangle with FP and Unghost with RP	Remove the noisy files from the bug-fixing commit and then add the extra files to it
DRURP	Detangle with RP and Unghost with RP	Remove the noisy files from the bug-fixing commit and then add extra files to it. The mentioned files are linked to the Mozilla repository using the RP strategy in both steps.

To address the problem of ghost commits (where the bug-introducing and bug-fixing commits do not share any common file as required by SZZ), we should add additional files in bug-fixing commits so that SZZ can trace their modification and identify bug-introducing commits. If these additional files are not modified in bug-fixing commits, then we can not apply the FP strategy as the intersection between the mentioned files and the modified files in the bug-fixing commits will be empty. Therefore, we leverage the bug discussion files and match them to the Mozilla code base using the RP strategy, resulting in the URP strategy shown in Table 3

**Fig. 5:**
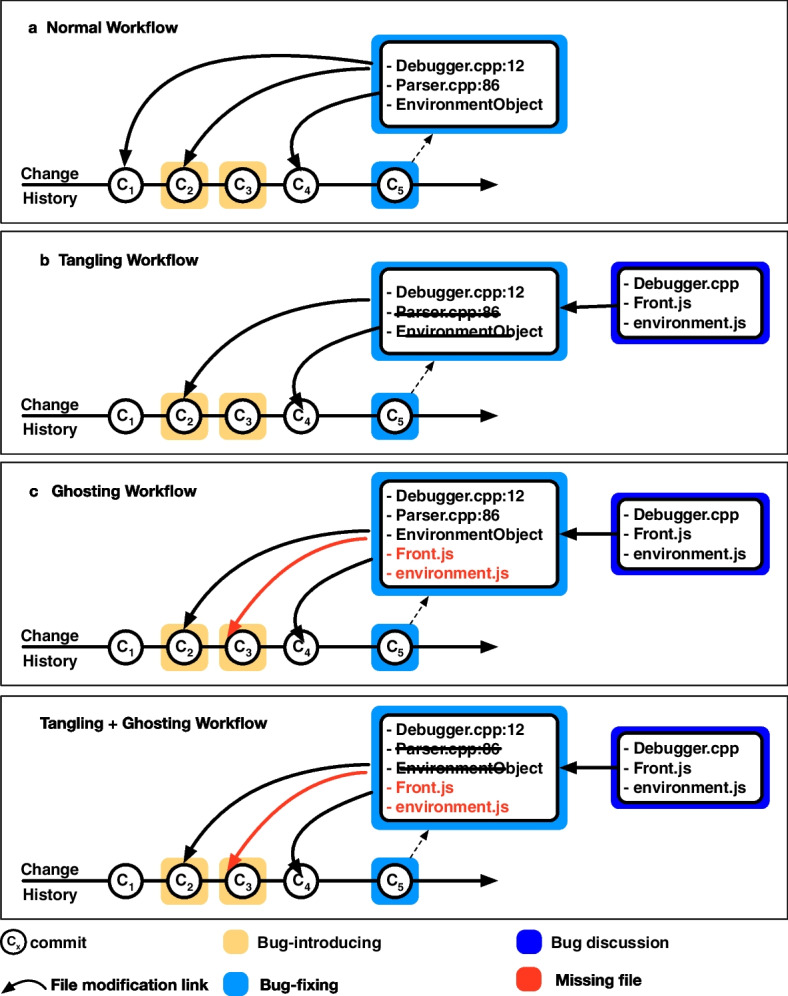
SZZ with (a) normal workflow, (b) tangling workflow, (c) ghosting workflow, and (d) tangling + ghosting workflow

We also test whether various parsing strategies applied in combination are more effective than applying them separately to address the problem of tangled commits and ghost commits. For example, if first removing noisy files from bug-fixing commit (using the DFP or DRP strategy) and then adding additional files (using the URP strategy) to it can be effective in handling the mentioned problems. Therefore, we combined DFP with URP and DRP with URP, resulting in DFURP and DFURP strategies , respectively, as shown in Table 3 and Fig. 5. In a nutshell, we keep files shared by the bug-fixing commits and bugs discussion to solve the tangling problem and files not part of bug-fixing commits but shared by the bug-introducing commits and bugs discussion to solve ghost commits.

Figure 5 shows numerous workflows and how files from bug discussion can help solve the problem of tangling commits (shown in detangling workflow) and ghost commits (shown in extrinsic workflow) compared to the normal workflow of SZZ.

To apply the approach of removing or adding files to bug-fixing commit, we adopted a recent implementation of SZZ provided by Rosa et al. (2021), *PySZZ*, as a baseline. We adapted their tool to have the option of executing git blame function only on the specified files rather than all files of a commit. Then, we selected five popular SZZ variations: B-SZZ, AG-SZZ, L-SZZ, R-SZZ, and MA-SZZ to test our hypothesis. We limited our scope to those variations since they do not leverage any language-specific features and, therefore, can be applied to every project (in the Mozilla ecosystem). For each SZZ variation, we deployed a dedicated virtual machine on an OpenStack cloud distribution and ran it on each strategy applied to the dataset, e.g., DRP, URP. Also, as another baseline, we created a normal version of the dataset without tangled or extrinsic workflow (‘Normal’ in Table 3 and ‘N’ in Table 9) to test SZZ variations.

Since SZZ works at the commit level and our dataset is based on PRs (where one PR can contain multiple commits); we created a commit-level version of our dataset by specifying files to be analyzed for each commit by SZZ. Table 2 describes the PRs and commits when the bugs have bug-introducing commits information, bug-fixing information, or both. If a commit does not modify any of the relevant files, it is discarded. If there is no overlap of files between the files modified in the bug-fixing commit and those mentioned in the discussion, we select the files from the ground truth prepared by the Mozilla developers to run SZZ on; this is to ensure that SZZ does not run on empty files. As mentioned earlier, SZZ requires a file shared by bug-fixing and bug-introducing commits to be able to link them. In our dataset, 7,554 bugs share at least one file among bug-fixing and bug-introducing commits thus they can be linked by existing SZZ. With our hypothesis, the new variant, FI-SZZ, can infer relevant files from bug discussions and thus can link in a total of 8,919 bugs as the files mentioned in bug discussion are modified in bug-introducing commits as shown in Table 4.

**Table 4:** Description of relevant bugs where SZZ can link bug-fixing commit to bug-introducing commit (FI-SZZ candidates) and sample bugs selected from the relevant bugs

Datasets	Bugs	Bug-fixing		Bug-introducing	
		PRs	Commits	PRs	Commits
Relevant bugs SZZ can link with our approach	8,919	7,509	48,781	8,919	60,191
Statistical significant sample	620	535	847	620	4,107

Computing SZZ for additional files may exponentially increase computational time due to the large number of files to match in a software repository and the limitation of the single-threaded git process (required for git annotate functions). Therefore, we extracted a statistically relevant sample of 620 randomly extracted bugs from 8,919 bugs. The size of this sample has been calculated using the same formula Triola et al. (2006) with a 99% of confidence level and a 5% margin of error with respect to the population size. In this sample set, we have 535 bugs that are labeled by developers (or ground truth). We leverage the script devised for *PySZZ* Rosa et al. (2021) to evaluate the results. We calculate the following information retrieval metrics Manning et al. (2010) that are used in the previous work as well Rosa et al. (2021):

$$\begin{aligned} recall = \frac{|correct \cap identified|}{|correct|} \end{aligned}$$

$$\begin{aligned} precision = \frac{|correct \cap identified|}{|identified|} \end{aligned}$$

$$\begin{aligned} F1 = 2 \cdot \frac{recall \cdot precision}{recall + precision} \end{aligned}$$

Where *identified* refers to the set of bug-introducing candidates commit-sets retrieved by SZZ, and *correct* refers to the set of bug-introducing commit-sets labeled by Mozilla developers in the ground truth. In the end, we tested whether the differences in recall and precision scores are statistically significant among the SZZ variations. We applied various statistical tests, such as the Shapiro-Wilk test and Pair T-Test Conover (1999). Specifically, we verified the distribution of recall and precision scores for each SZZ variation with the Shapiro-Wilk test. We found that these distributions follow a normal distribution. Then, we applied the statistical Pair T-Test using SciPy^8^ library to demonstrate the relevance of our parsing strategies and measure their effect size.

## Results

### $RQ_1$: Rationales behind File Discussion

For $RQ_1$, we analyzed the rationales of why developers mention files in bug reports. From the preliminary analysis, we identified frequent cases where developers reference some testing files or links to wikis in the discussion. Not all these references were actually posted by developers. For example, wikis were often posted by bots. We also observed that the files were referenced in a specific format, *e*.*g*., with line numbers or without extensions. Informed from these findings, we adapted the file extraction technique accordingly. Also, the preliminary analysis helped us in formulating the initial draft of the taxonomy of rationales, *e*.*g*., System Dumps, Bug dependency. In the next step, we validated and extended this taxonomy with a larger sample of 369 bugs. Based on the analysis of 369 bugs, we obtained the eleven categories described in Table 5.

**Table 5:** Resulting taxonomy of the rationales behind the files mentioned in bug discussions

Category	Description	#Bugs
*System dumps*	The files appear in a crash report or a system log	240
*Bug description*	Developers mention the files in describing the bug in details; such files got impacted in the bug, might have caused the bug, or can fix the bug	127
*Artifact reference*	Developers mention the files in the title of a bug, an attachment, or a commit message	120
*Bug reproducibility*	Developers mention the files that can help reproduce the bug	49
*Solution draft*	Developers propose a modification to the files to draft a bug solution	49
*Indirect resolutions*	Developers mention the files in the bug that is fixed by backout, missing mapping from Thunderbird, or no fix decision	41
*Link to extra files*	Developers link to the online source of the file, to wiki files, to additional files to reproduce the bug, or to failure logs	19
*Bug dependency*	Developers mention the files that partly caused or fixed a dependent bug	18
*Code review*	Developers mention the files in a code review round	6
*Code snippet*	The files appear in a code snippet discussed by developers	4

Most file names appearing in bug discussions are classified as *System dumps*: they are part of crash reports, stack traces, and logs, usually provided by bots that monitor the continuous integration and development (CI/CD) pipeline.

Another common reason developers mention files is that they are trying to understand the bug and speculate on the files that might have caused it or can help fix it (*Bug description* category in Table 5). For example, in one of the bugs [*Bug:1739924*], the developer points out an unexpected file “We have not touched StringBundle in years as it is on the path to being deprecated.” In another instance [*Bug:1620314*], a developer speculated on the probable reason for the bug and suggested modification in various mentioned files. However, we found that developers do not always mention the names of the files but rather the buggy code elements or snippets from them. For such cases, involving additional code element information in automated defect prediction techniques can help find the root cause of the bug quicker.

One of the initial steps of the bug life cycle is to reproduce the bug. We observed that developers spend a significant effort in reproducing the bugs (the third most frequent category, *Bug reproducibility* in Table 5). Such files can be external files that are specific to the user (local files in the user system), test case files that detect the bug, or additional files that the user thinks can help reproduce the bug step-by-step.

Once a bug is reproduced, developers discuss various solutions (*Solution draft*) to resolve the bug. They often provide modifications in a code snippet of the file but do not always mention the corresponding file names. All these suggested drafts in the bug discussion do not become directly part of the final fix. Other developers review these drafted solutions and propose further changes (*code review* category). For example, the developer reviews the code in the bug discussion of [*Bug:1623285*] and points out that the bug was overlooked in the code review. Such instances indicate developers care about the code review to maintain the code quality.

Developers do not always mention the file names related to fixing bugs; they often provide links to these files (*Link to extra files* category) so that other developers can directly access the files within the bug discussion to understand and fix the bug quickly. These links direct to files on other sources, such as GitHub, Mozilla source code browser, and Wikis.^9^ We also found the files are mentioned in various artifacts (*Artifact reference*) as well, *e*.*g*., title of a bug, a bug-fixing commit, or an attachment. Also, in some cases, the files were not directly involved in fixing the bug but rather part of other bugs that the current bug depended on (*bug dependency*). For example, the developer refers to another bug where the files are discussed [*Bug:1770165*], or the developer fixes the bug by fixing a dependent file in [*Bug:1538049*].

For the remaining cases of files mentioned in bug discussions, we defined the *Indirect resolutions* category shown in Table 5. This category covers the bug discussions that are reverted back (backout) to fix the bug, or no decision has yet been taken on fixing it. For example, in [*Bug:1734984*], a changeset caused the failure in a file (mentioned in the bug discussion); however, the bug is resolved by reverting that changeset rather than touching the file. Therefore, the bugs in this category have many files mentioned in bug discussions.

### $RQ_2$: Linking Referenced Files to Mozilla Ecosystem

As the files mentioned in bug discussions can be part of the files modified in bug-introducing commit(s) or bug-fixing commit(s) or they might not be. Recognizing the files that truly belong to bug-introducing and bug-fixing commits can bolster the efficiency of the SZZ algorithm as it requires at least one shared file between the bug-introducing or bug-fixing commit(s) to establish a connection. In this RQ, we linked the files mentioned in the bug discussion to the files modified in bug-fixing or bug-introducing commits at the PR level. Table 6 shows the distribution of bugs in which the files mentioned in bug discussion are shared with a bug-fixing PR (FFC), bug-introducing PR (FBC), both (FFBC) and neither of them (FNC) and in what proportion do they exist in our overall dataset and sample dataset.

**Table 6:** Overview of sample bug discussion with respect of each case

Case	Case description	#Bugs	% of Dataset	#Sample
FFC	File references shared with bug-fixing PR only	995	10%	37
FBC	File references shared with bug-introducing PR only	1,179	13%	48
FFBC	File reference shared with both	4,376	47%	173
FNC	File reference shared with neither	2,731	30%	111
	With at least one file reference in bug discussion	9,281	100%	369

We found that developers discussed at least one file in 74.41% of the bugs (9,281 bugs), and in 70.47% of those bugs (6,550 bugs), one of the discussed files is part of the bug-introducing or bug-fixing PR, or both. SZZ requires at least one file to be shared between the bug-fixing and bug-introducing PR to be able to link them, and that file should not have only added lines (as SZZ can not trace edits on such files). Fulfilling its requirements, SZZ in our dataset can potentially link 60.56% of bug-fixing PRs to bug-introducing PRs (7,554 records out of 12,472). However, SZZ still fails to link 40% of bug-fixing PR to bug-introducing PR as there is no shared file between them.

**Table 7:** The files mentioned for various rationales and shared with bug-fixing commits (FFC), bug-introducing commits (FBC), both commits (FFBC), and neither (FNC)

Rationale	#FFC	#FBC	#FFBC	#FNC
*System dumps*	19	38	118	64
*Bug description*	21	13	72	21
*Artifact reference*	15	21	79	5
*Bug reproducibility*	3	5	11	30
*Solution draft*	4	5	21	19
*Indirect resolutions*	2	10	4	25
*Link to extra files*	2	3	3	11
*Bug dependency*	0	9	3	6
*Code review*	0	0	1	5
*Code snippet*	0	0	2	2

Not all of the files mentioned in bug discussions are modified in commits of our interest. Table 7 shows four categories of bugs for the sample dataset where the mentioned files are modified only in bug-fixing commit (FFC), bug-introducing commit (FBC), both (FFBC), or neither (FNC). From our manual analysis of 369 bugs shown in Table 7, we can see that not all of these mentioned files (in dumps) are valuable for resolving the bug. Among the files mentioned in bug discussions and matched in the Mozilla system (according to the matching strategies), we observed that in 25% of the cases (FNC), these mentioned files are not involved in resolving bugs (or are not modified in the bug-introducing or bug-fixing commits) while in 75% of the cases (FFC, FBC, and FFBC), these files help identify or fix the bug. The files that could be relevant to SZZ tend to be on the top of the stack trace.

In RQ_1_, we found various reasons why developers mention files in bug reports. In this RQ, we identified frequent cases in which the files are discussed in the bug reports, but they are not modified in either bug-fixing or bug-introducing commits (FNC cases of 111 bug reports). Such files in the future can be utilized to further solve the problem of ghost commits, where the bug-introducing and bug-fixing commits do not share any file or the changes that cause or fix the bug lie outside of the repository.

**Table 8:** Description of sample 620 bugs with the files referenced in their bug reports and matched in the Mozilla ecosystem

Workflow	Strategy	Bugs	# Files mentioned in Reports	# Matched Files in Mozilla
Tangled (RQ3)	DFP	620	4,175	1,071
	DRP	620	4,175	1,484
Ghost (RQ4)	URP	620	4,175	2,564
Tangled + Ghost	DFURP	620	4,175	1,910
	DRURP	620	4,175	2,323

With our approach, we can add the files mentioned in bug discussions in bug-fixing commits and thus can target an additional 1,336 bugs or a total of 8,990 bugs. Specifically, we can target 3,600 bugs: we can remove changes possibly unrelated to the fix for 2,164 bugs or bug-fixing PRs to reduce the noise of *tangled commits* ($RQ_3$) as the bug discussion mentions at least one file that is also modified in bug-fixing PR. We can add additional files extracted from bug discussions to the respective bug-fixing PRs for 1,436 *ghost* commits ($RQ_4$) as the bug discussion mentions at least one file that is also modified in the bug-introducing PR. Overall, our approach should impact 28.86% (3,600 bugs) of the dataset as in the remaining cases, *i*.*e*., 71.14% (8,872 bugs), a file mentioned in the bug discussion is not part of either of bug-introducing or bug-fixing PRs (FNC). For the sampled 620 bugs for RQ3 and RQ4 as shown in Table 8, we extracted the files referenced in their bug discussions and matched the files in the Mozilla ecosystem based on various parsing strategies (as shown in Table 3).

### $RQ_3$: Mitigating Tangled Commits

Once we identified the files being mentioned in bug discussions and being modified in commits of our interest, we investigated whether these files could be useful to detangle commits and identify bug-introducing commits.

Table 9 shows the results for the selected SZZ variations, *e*.*g*., B-SZZ, AG-SZZ for various detangled datasets formulated using different file parsing strategies, *i*.*e*., the normal version of the dataset (shown in the *N* column), the dataset prepared using the FP strategy (shown in the *FP* column), and the dataset prepared using the RP strategy (shown in the *RP* column). The results are computed for various parsing strategies on the representative sample of 620 bugs, where 535 bugs are labeled by Mozilla developers (ground truth).

Since the input of SZZ is filtered using the files mentioned in a bug discussion, we can only improve the precision of SZZ by discarding noisy files from the computation. Table 9 shows that for all SZZ variations (except L-SZZ), we lose from 1 to 4 percentage in recall points for an improvement in the precision of 2 to 4 percentage points compared to the normal case (‘N’). The DFP strategy (detangled with bug-fixing parsing) is more effective than DRP, as shown in the DFP column in F-measure, increasing the F-score of all SZZ versions except R-SZZ. With our approach, even the noisy versions of SZZ, *i*.*e*., B-SZZ, AG-SZZ, and MA-SZZ significantly improve their precision: over 300 false positive records from their normal version of the SZZ (denoted by the N column in Table) have been discarded in the DFP strategy (shown in *DFP* column in Identified). For instance, R-SZZ obtains a minor improvement in precision despite the algorithm’s overall performance being unchanged (represented by F-Score). The correctly identified bugs are almost identical for each variation and parsing strategy.

**Fig. 6:**
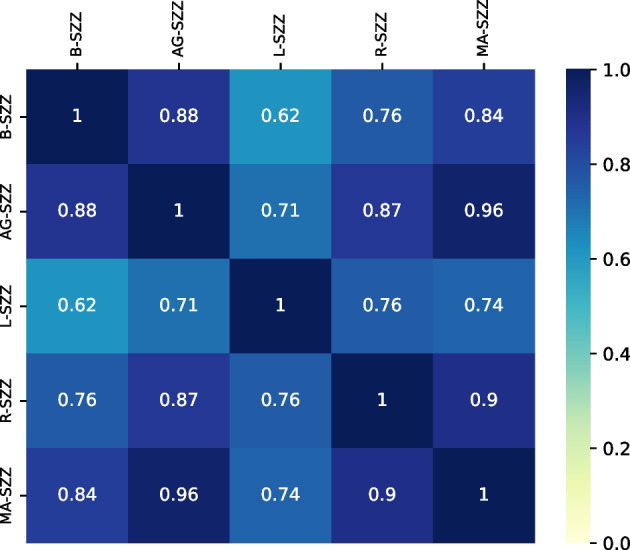
Overlap between SZZ variations’ when the DFP strategy is applied

Figure 6 shows the agreement of SZZ variants on the DFP strategy. It reveals a significant overlap between the variants as 9 out of 10 comparisons share more than 70% in the correctly identified bugs. This highlights that the DFP strategy (*i*.*e*., keeping the only files in the bug-fixing commit that are mentioned in the bug discussion) does not undermine the number of bugs correctly linked by SZZ. While it reduces the number of identified bug-introducing commits, it also produces more accurate links from bug-fixing commits to bug-introducing commits. Studies have shown various effects of false positives on developers, such as developers having to spend more time reviewing false positives and lacking trust in static analysis tools and defect prediction models Imtiaz et al. (2019); Wan et al. (2020). Having fewer but more accurate results in the case of identifying bug-introducing commits can help developers better trust the tools and techniques based on them.

**Table 9:** SZZ ’s performance with detangling strategies DFP and DRP (Sample Bugs = 620, Correct (labeled by developers) = 535)

Variations	Identified			Corr. $\cap$ Ident.			Recall			Precision			F-Measure		
	N	DFP	DRP	N	DFP	DRP	N	DFP	DRP	N	DFP	DRP	N	DFP	DRP
B-SZZ	**1,383**	1,056	1,212	242	220	236	**0.45**	0.41	0.44	0.17	**0.21**	0.19	0.25	**0.28**	0.27
AG-SZZ	**1,333**	1,013	1,136	217	201	213	**0.41**	0.38	0.40	0.16	**0.20**	0.19	0.23	**0.26**	0.25
L-SZZ	**503**	446	481	147	142	147	0.27	0.27	0.27	0.29	**0.32**	0.31	0.28	**0.29**	**0.29**
R-SZZ	**513**	455	492	185	174	182	**0.35**	0.33	0.34	0.36	**0.38**	0.37	0.35	0.35	0.35
MA-SZZ	**1,439**	1,092	1,238	**209**	193	206	**0.39**	0.36	**0.39**	0.15	**0.18**	0.17	0.21	**0.24**	0.23

The bold values denote the largest value achieved by one of the strategies

**Table 10:** SZZ ’s performance with both detangling and unghosting strategies DFURP and DRURP

Variations	Identified			Corr. $\cap$ Ident.			Recall			Precision			F-Measure		
	N	DFURP	DRURP	N	DFURP	DRURP	N	DFURP	DRURP	N	DFURP	DRURP	N	DFURP	DRURP
B-SZZ	1,383	1,057	1,228	242	220	236	**0.45**	0.41	0.44	0.17	**0.21**	0.19	0.25	**0.28**	0.27
AG-SZZ	1,333	1,016	1,148	217	201	213	**0.41**	0.38	0.40	0.16	**0.20**	0.19	0.23	**0.26**	0.25
L-SZZ	503	446	481	147	142	147	0.27	0.27	0.27	0.29	**0.32**	0.31	0.28	**0.29**	**0.29**
R-SZZ	513	455	493	185	174	182	**0.35**	0.33	0.34	0.36	**0.38**	0.37	0.35	0.35	0.35
MA-SZZ	1,439	1,097	1,249	**209**	193	206	**0.39**	0.36	**0.39**	0.15	**0.18**	0.16	0.21	**0.24**	0.23

(Sample Bugs = 620, Correct (labeled by developers) = 535). The bold values denote the largest value achieved by one of the strategies

We performed various statistical tests, such as the Shapiro-Wilk test, to verify the distribution of precision and recall and the statistical Paired T-Test to find the improvement brought by our file parsing strategies. Table 11 shows the p-values for the precision and recall values on our dataset with the normal approach, and our approach (the DFP strategy) applied (indicated by the *N vs. DFP* column and *N vs. DRP* column) for selected SZZ variants. We can see that the p-value for the precision of the normal approach compared to the DFP strategy for R-SZZ is significant (< 0.05),   i.e., $\alpha = 0.02$. Similarly, the precision of the normal approach compared to the DRP strategy for B-SZZ and AG-SZZ is below the standard significant size or p-values are significant, *i*.*e*., $\alpha = 0.01$ and $\alpha = 0.04$ respectively. Thus, we can say that the parsing strategies impact the precision of R-SZZ, B-SZZ, and AG-SZZ.

In the case of Recall for both strategies (*N vs. DFP* and *N vs. DRP*), p-values are above the standard significant size, $\alpha = 0.05$, except for R-SZZ in *N vs. DFP*, thus showing that the parsing strategies are not so effective for improving the recall of SZZ variations except for R-SZZ. To measure the effect of parsing strategies over the normal version, we use a Paired T-test. Table 11 shows that the DFP strategy has a larger effect on the improvement of precision for the majority of the SZZ variations.

**Table 11:** Paired T-Test for Recall and Precision of Detangling options (file parsing strategies (DFP or DRP) compared to the normal approach with *effect size* and *p-value*

Variations	N vs DFP				N vs DRP			
	Precision		Recall		Precision		Recall	
	*effect*	*p-value*	*effect*	*p-value*	*effect*	*p-value*	*effect*	*p-value*
B-SZZ	**4.75**	**2.51e-06**	1.09	0.27	**2.46**	**0.01**	-0.43	0.66
AG-SZZ	**4.02**	**6.50e-05**	-0.26	0.79	**2.00**	**0.04**	-0.72	0.46
L-SZZ	1.66	0.09	1.20	0.22	0.00	1.00	-0.90	0.36
R-SZZ	**3.02**	**0.02**	**2.05**	**0.04**	1.73	0.08	0.75	0.45
MA-SZZ	**4.02**	**6.50e-05**	-0.03	0.97	1.73	0.08	-1.12	0.26

### $RQ_4$: Mitigating Ghost Commits

We included the additional files mentioned in the bug discussion in bug-fixing commits to investigate whether they could mitigate the ghost commits. Table 12 shows the performance of SZZ variations (shown in the *variations* column), with the normal approach (shown in the *N* column) and the URP approach.

**Table 12:** SZZ ’s performance with unghosting option (normal approach and URP approach) ($Bugs = 620, Correct = 535$)

Variations	Identified		Corr. $\cap$ Ident.		Recall		Precision		F-Measure	
	N	URP	N	URP	N	URP	N	URP	N	URP
B-SZZ	1,383	**1,386**	242	242	0.45	0.45	0.17	0.17	0.25	0.25
AG-SZZ	1,333	1,333	217	217	0.41	0.41	0.16	0.16	0.23	0.23
L-SZZ	503	**508**	147	**148**	0.27	**0.28**	0.29	0.29	0.28	0.28
R-SZZ	513	**519**	185	185	0.35	0.35	0.36	0.36	0.35	0.35
MA-SZZ	1,439	**1,440**	209	209	0.39	0.39	0.15	0.15	0.21	0.21

The bold values denote the largest value achieved by one of the approaches

Table 12 shows that linking additional files is counter-productive: the bug-introducing commit sets retrieved by SZZ is slightly increased but did not improve the recall substantially. Only for L-SZZ, the recall is increased by 0.01, without significantly impacting the F-Score. Table 10 reports the results for the combined approaches for detangling and extrinsic workflows, *i*.*e*., DFURP and DRURP presented in Table 3. Even in this case, extrinsic reference support does not significantly impact: SZZ performances are similar to those reported in Table 9.

## Discussion and Future Work

Previous work has shown various approaches to link bug-fixing commits to bug-introducing commits for VCS, as accurate links will lead to more reliable datasets and eventually to better defect prediction models. In our study, we investigated whether the files mentioned by developers in the bug discussion can be leveraged to identify the links to bug-introducing commits. Although our approach showed some promise in helping tackle the problem of tangled commits, it is ineffective in solving the problem of ghost commits. One approach to improve could be to infer the external files from other sources. In our manual analysis of the bug reports, we observed that developers discuss particular code elements without mentioning the named of the files in which they are located. The current variations of SZZ leverage the files (modified in bug-fixing commits) to trace bug-introducing commits rather than the code elements. Inferring these file names based on the discussed code elements or snippets can further hint towards the files involved in causing or fixing the bug. However, to do so, matching the code elements or code snippets (mentioned in bug discussions) in the whole project ecosystem is currently challenging due to the large potential candidate space and prevalent code clones in the ecosystem. Future automated bug prediction techniques can leverage these additional code elements or snippets to precisely identify the root cause of the bug. Although the solutions based on code elements would be language-specific and would work on specific SZZ versions, we hypothesize that they can further recover the links to bug-introducing commits if they can be used to infer the relevant files. Such solutions can be combined with more sophisticated static analysis techniques, such as analyzing the correlation between the files involved in bug-introducing and bug-fixing commits and the other external files co-changed with them. Recently, ChatGPT has been used for various SE tasks White et al. (2023); we expect that ChatGPT can be used in future to further extract the relevant parts (code elements, file references) from the bug discussion and can help improve SZZ in identifying buggy code.

In addition to using bug discussions, one can use other artifacts, such as discussions from the mailing lists and communication channels (slack or Wikis), to further add and verify the file links. Although we expect such approaches to be useful for finding external files, they require heavy computational load due to the use of git to run SZZ. To speed up the process, a new way to store the development information is first required to parallelize SZZ computations.

Bug reports also show other types of information developers communicate. For example, we observed in [*Bug:1739784*] where developers discuss who should own the bug to fix it. Although such information can not be directly used by SZZ (as it uses the file modified in bug-fixing commits to infer bug-introducing commits), future work can find instances of this information in bug discussions and leverage them to improve the bug assignment techniques. Researchers have explored the effect of ownership Bird et al. (2011) and showed its importance in maintaining high-quality software; Even though Bugzilla has the feature to assign the bug to a user, developer discussion in the bug reports hints that ownership can still be unclear within a team and can require further discussion. In another case [*Bug:1761233*], the developer referred to another expert developer to find the root cause of the bug. Such instances show the need to develop advanced techniques to automatically find the right owner to fix the bug and save other developers time quickly.

In our study, we saw in [*Bug:1625151*] that developers are interested in having advanced static analysis tools to perform a high-level analysis of the bugs to save time. In our current approach, we have not considered any knowledge of developers who discussed these files in the issue tracker or those who modify the files in bug-fixing commits. Future studies can explore what other communication channels these developers use and can leverage those sources to further improve SZZ.

In our current version of SZZ, we have used the annotate function (git blame) on the changed lines in bug-fixing commits, which identifies the bug-introducing commits based on when these changed lines were lastly modified. However, there is a possibility that bug-introducing commits are not always in the most recent change but rather hidden further back in the change history. Nguyen et al. (2016) have shown that the bug-introducing commits are often the initial code changes for specific bugs, *e*.*g*., vulnerabilities. Based on their work, Bao et al. (2022) proposed an improved version of SZZ, V-SZZ, to identify bug-introducing for these bugs.. Future work can consider other variations to explore the change history of Mozilla further.

## Threats to Validity

### Construct Validity

The main concern for the construct validity is related to the measurements used in the analysis we selected. Mozilla follows a rather different process to many popular development systems, *rebase* strategy, to merge a set of commits to the main branch. Consequently, all commits in the set appear as a single branch history. Also, the intermediate commits can be tracked without losing authorship information, which is necessary for SZZ to find the bug-introducing commits. Our approach might not directly work for the systems that follow the *squash* strategy (the last commit supersedes the previous commits) to merge commits to the main branch.

To extract file paths from bug discussions available in the issue tracker, we used an automated script (used by previous work as well) Petrulio et al. (2022). To further verify the correctness of file paths, two authors manually verified a sample of the file paths through a preliminary analysis to discard irrelevant links (such as wiki links posted by bots).

To filter (to include or exclude files) files from bug-fixing commits based on the files referenced in the bug discussion, we used a custom file path-matching criteria with a threshold of 0.5 (relaxed parsing strategy). We chose this threshold based on manual analysis and iterative process. We also tested with a strict parsing strategy with a threshold of 0.8 and found that the strict parsing strategy did not filter any files from bug discussions for SZZ input: files in bug discussion are rarely mentioned with the correct or full path, making the comparison with modified files in bug-fixing commits rather difficult. Additionally, if a file underwent renaming, our approach can handle such case as we use a custom file-path matching approach based on folder matching and with a relaxing threshold of 0.5.

The next concern is the scope of ghost commits. Recent studies show that the bugs can be due to the changes recorded in VCS (known as intrinsic bugs) or due to the changes that happened outside of the repository (known as extrinsic bugs) Rodríguez-Pérez et al. (2018). We considered the ghost commits for intrinsic bugs as the latter case (changes happening outside of the repository) is not the direct responsibility of the Mozilla developers Herbold et al. (2022b). Thus, we focused on the bugs whose origin still lies in the VCS but whose fix happened outside of the VCS. In such cases, the fix changes are not captured in bug-fixing commits; therefore, the heuristics of SZZ can not identify bug-introducing commit. We provided SZZ additional files from bug discussion to the bug-fixing commit to trace its bug-introducing commits.

The last concern is the ground truth and the evaluation approach used in our work. We used precision and recall measures, used in the previous work as well Rosa et al. (2021) to quantify the accuracy of our linking approach on the Mozilla dataset. This dataset of PRs is labeled by Mozilla developers, providing us with reliable ground truth, and it is used in previous works as well Petrulio et al. (2022).

### Internal Validity

Generally, performing manual analysis introduces subjectivity in the process. To reduce the chances of choosing unrepresentative samples, we used a random sampling approach. We followed a standard approach of selecting a statistically significant sample, limiting the error to 5% for a confidence level of 95%. Similarly, we selected the statistically significant sample of 620 bugs to run selected SZZ variations, limiting the error to 5% for a confidence level of 99%.

To reduce the effect of biases and subjectiveness in the manual analysis and in formulating the taxonomy of rationales, two authors got involved in the analysis. They independently analyzed their assigned samples not to influence each other classification. In 94% of the cases, the authors agreed on the rationales (completely or partially). The disagreement cases (nearly 6%) were often due to different formats of reporting files in *System dumps*, *bug description*, and others. We mutually discussed every case of disagreement to finalize the categories.

### External Validity

The main concern for the external validity is the generalization of our results. We sourced bug discussions from the Mozilla ecosystem for the reasons mentioned in Sections 3.2 and 1. Mozilla employs an open-source issue tracker system, *e*.*g*., Bugzilla. Since our approach infers the files based on NLP techniques, the file names can be inferred from other bug discussion platforms such as JIRA. Similarly, our file parsing strategy, being independent of language-specific choice, can also be used to match the files mentioned in the discussion with the files modified in the change history.

Another concern is our dataset is linked at the commit-set (PR) level, whereas SZZ is devised to work at the commit level. This can introduce some noise in SZZ results; for example, the squash commits can lead to loss of authorship information or their individual commits. However, Mozilla relies on the rebase strategy, which makes the version history linear and preserves the author’s information. Also, the results achieved by our previous work Petrulio et al. (2022) in folding and unfolding commits at the PR level for SZZ are in line with the ground truth by Mozilla developers. Also, many PRs are composed of a single commit, thus further narrowing down the scope of potential noise by SZZ.

Various organizations can choose various platforms to host their source code, bug reports, bug discussion *etc.*. Although different ecosystems, such as Google, Apache *etc.* can use different systems of issue trackers or version control systems, developer discussion on bugs remains similar on these platforms in natural languages. We selected the dataset and ecosystem from Mozilla due to its diversity in terms of languages and domains. On the one hand, our choice of the dataset complements the existing datasets in defect prediction; on the other hand, it can be generalized to other ecosystems due to the file parsing strategy being independent of language-specific choice.

### Conclusion Validity

To ensure the relationship between the treatment and the outcome, we manually analyze a sample of bug discussion and several relevant statistical tests, such as measuring the data distribution using the Shapiro-Wilk test and paired T-test to compare the parsing strategies on SZZ variations.

## Conclusions

Researchers have conducted various empirical studies to understand the cause and impact of poor software quality, *e*.*g*., identifying the buggy code or code changes that introduced a bug. Several approaches, *e*.*g*., SZZ, are established to link bug reports to bug-fixing code and eventually to buggy code. However, such approaches are inaccurate or biased due to the lack of ground truth (by developers) and the various assumptions they hold. The problems of tangled commits and ghost commits are examples of problems that undermine these assumptions.

Developers discuss bugs in bug reports and embed various kinds of information. We analyze the content of bug reports and investigate whether the content can enhance the performance of the SZZ in identifying bug-introducing commits more accurately. We found that including files mentioned in the bug discussion can enhance the effectiveness of SZZ in identifying bug-introducing commits by handling tangled commits. Our strategy, however, is still ineffective in handling the problem of ghost commits. We provide the dataset called *RoTEB*, comprised of 12,472 bugs from Mozilla (the links established by Mozilla developers), serving as a resource for further inquiries into software quality criteria.

## Data Availability

The dataset prepared and analyzed in the study and the resultant software (a version of SZZ, and code used to analyze the dataset) are available as a part of our replication package at the following link Anonymous (2023): https://doi.org/10.5281/zenodo.11484723

## Footnotes

1 https://bugzilla.mozilla.org/show_bug.cgi?id=1666140

2 https://www.mozilla.org/en-GB/

3 https://www.mozilla.org/en-US/

4 https://wiki.mozilla.org/BMO/UserGuide/BugFields

5 https://github.com/PR-research-data-tools/RP-remodeling-szz-discussions/tree/main/RQ2-RQ3-RQ4/SZZ-evaluation/preliminary_analysis.ipynb

6 https://github.com/PR-research-data-tools/RP-remodeling-szz-discussions/tree/main/RQ2-RQ3-RQ4/SZZ-evaluation/preliminary_analysis.ipynb

7 https://wiki.mozilla.org/BMO/UserGuide/BugFields

8 https://scipy.org/

9 Please note that we only considered the files that are part of a direct link (HTML link), and if files are mentioned on the resultant page of the link rather than on the bug page, we ignored such cases.

## References

[CR1] Anonymous (2023) Replication package for ‘On refining the szz algorithm with bug discussion data’.10.5281/zenodo.11484723

[CR2] Bao L, Xia X, Hassan AE, Yang X (2022) V-szz: automatic identification of version ranges affected by cve vulnerabilities. In Proceedings of the 44th international conference on software engineering, pp 2352–2364

[CR3] Bird C, Nagappan N, Murphy B, Gall H, Devanbu P (2011) Don’t touch my code! examining the effects of ownership on software quality. In Proceedings of the 19th ACM SIGSOFT symposium and the 13th European conference on Foundations of software engineering, pp 4–14

[CR4] Bludau P, Pretschner A (2022) Pr-szz: How pull requests can support the tracing of defects in software repositories. In 2022 IEEE international conference on software analysis, evolution and reengineering (SANER), pp 1–12. IEEE

[CR5] Braun V, Clarke V (2006) Using thematic analysis in psychology. Qual Res Psychol 3(2):77–101 10.1191/1478088706qp063oa

[CR6] Castelluccio M (2021) Bugbug. 10.5281/zenodo.4911346

[CR7] Chen B, Jiang ZM (2019) Extracting and studying the logging-code-issue-introducing changes in Java-based large-scale open source software systems. Empir Softw Eng 24:2285–2322 10.1007/s10664-019-09690-0

[CR8] Coleman G, O’Connor R (2007) Using grounded theory to understand software process improvement: A study of irish software product companies. Inf Softw Technol 49(6):654–667 10.1016/j.infsof.2007.02.011

[CR9] Conover WJ (1999) Practical nonparametric statistics, vol 350. John Wiley & Sons

[CR10] Da Costa DA, McIntosh S, Shang W, Kulesza U, Coelho R, Hassan AE (2016) A framework for evaluating the results of the szz approach for identifying bug-introducing changes. IEEE Trans Softw Eng 43(7):641–657 10.1109/TSE.2016.2616306

[CR11] Davies S, Roper M, Wood M (2014) Comparing text-based and dependence-based approaches for determining the origins of bugs. J Softw Evol Process 26(1):107–139 10.1002/smr.1619

[CR12] Fan Y, Xia X, Da Costa DA, Lo D, Hassan AE, Li S (2019) The impact of mislabeled changes by szz on just-in-time defect prediction. IEEE Trans Softw Eng 47(8):1559–1586 10.1109/TSE.2019.2929761

[CR13] Gousios G, Storey MA, Bacchelli A (2016) Work practices and challenges in pull-based development: The contributor’s perspective. In Proceedings of the 38th international conference on software engineering, pp 285–296

[CR14] Herbold S, Trautsch A, Ledel B, Aghamohammadi A, Ghaleb TA, Chahal KK, Bossenmaier T, Nagaria B, Makedonski P, Ahmadabadi MN, Szabados K et al (2022) A fine-grained data set and analysis of tangling in bug fixing commits. Empir Softw Eng 27(6):125 10.1007/s10664-021-10083-5

[CR15] Herbold S, Trautsch A, Trautsch F, Ledel B (2022) Problems with szz and features: An empirical study of the state of practice of defect prediction data collection. Empir Softw Eng 27(2):42 10.1007/s10664-021-10092-4

[CR16] Herzig K, Just S, Zeller A (2016) The impact of tangled code changes on defect prediction models. Empir Softw Eng 21(2):303–336 10.1007/s10664-015-9376-6

[CR17] Imtiaz N, Rahman A, Farhana E, Williams L (2019) Challenges with responding to static analysis tool alerts. In 2019 IEEE/ACM 16th international conference on mining software repositories (MSR), pp 245–249. IEEE

[CR18] Kalliamvakou E, Gousios G, Blincoe K, Singer L, German DM, Damian D (2014) The promises and perils of mining github. In Proceedings of the 11th working conference on mining software repositories, pp 92–101

[CR19] Kamei Y, Shihab E, Adams B, Hassan AE, Mockus A, Sinha A, Ubayashi N (2012) A large-scale empirical study of just-in-time quality assurance. IEEE Trans Softw Eng 39(6):757–773 10.1109/TSE.2012.70

[CR20] Kawrykow D, Robillard MP (2011) Non-essential changes in version histories. In 2011 33rd International conference on software engineering (ICSE), pp 351–360. IEEE

[CR21] Kim S, Zimmermann T, Pan K, James Jr E, et al (2006) Automatic identification of bug-introducing changes. In 21st IEEE/ACM international conference on automated software engineering (ASE’06), pp 81–90. IEEE

[CR22] Kochhar PS, Tian Y, Lo D (2014) Potential biases in bug localization: Do they matter? In Proceedings of the 29th ACM/IEEE international conference on Automated software engineering, pp 803–814

[CR23] Koru AG, Liu H (2005) Building effective defect-prediction models in practice. IEEE Softw 22(6):23–29 10.1109/MS.2005.149

[CR24] Krishna R, Pryor D, Menzies T (2015) The promise repository of empirical software engineering data

[CR25] Lenarduzzi V, Palomba F, Taibi D, Tamburri DA (2020) Openszz: A free, open-source, web-accessible implementation of the szz algorithm. In Proceedings of the 28th international conference on program comprehension, pp 446–450

[CR26] Manning C, Raghavan P, Schütze H (2010) Introduction to information retrieval. Nat Lang Eng 16(1):100–103

[CR27] Mills C, Parra E, Pantiuchina J, Bavota G, Haiduc S (2020) On the relationship between bug reports and queries for text retrieval-based bug localization. Empir Softw Eng 25(5):3086–3127 10.1007/s10664-020-09823-w

[CR28] Navarro G (2001) A guided tour to approximate string matching. ACM Comput Surv (CSUR) 33(1):31–88 10.1145/375360.375365

[CR29] Neto EC, Da Costa DA, Kulesza U (2018) The impact of refactoring changes on the szz algorithm: An empirical study. In 2018 IEEE 25th International Conference on Software Analysis, Evolution and Reengineering (SANER), pp 380–390. IEEE

[CR30] Neto EC, Da Costa DA, Kulesza U (2019) Revisiting and improving szz implementations. In 2019 ACM/IEEE international symposium on empirical software engineering and measurement (ESEM), pp 1–12. IEEE

[CR31] Nguyen VH, Dashevskyi S, Massacci F (2016) An automatic method for assessing the versions affected by a vulnerability. Empir Softw Eng 21:2268–2297 10.1007/s10664-015-9408-2

[CR32] Petrulio F, Ackermann D, Fregnan E, Calikli G, Castelluccio M, Ledru S, Denizet C, Humphries E, Bacchelli A (2022) Szz in the time of pull requests. arXiv:2209.03311

[CR33] Ramsauer R, Lohmann D, Mauerer W (2019) The list is the process: Reliable pre-integration tracking of commits on mailing lists. In 2019 IEEE/ACM 41st international conference on software engineering (ICSE), pp 807–818. IEEE

[CR34] Rani Pooja, Panichella Sebastiano, Leuenberger Manuel, Ghafari Mohammad, Nierstrasz Oscar (2021) What do class comments tell us? an investigation of comment evolution and practices in pharo smalltalk. Empir Softw Eng 26(6):112 10.1007/s10664-021-09981-5

[CR35] Ray B, Hellendoorn V, Godhane S, Tu Z, Bacchelli A, Devanbu P (2016) On the “naturalness” of buggy code. In Proceedings of the 38th International Conference on Software Engineering, pp 428–439

[CR36] Rezk C, Kamei Y, Mcintosh S (2021) The ghost commit problem when identifying fix-inducing changes: An empirical study of apache projects. IEEE Trans Softw Eng

[CR37] Rodríguez-Pérez G, Robles G, Serebrenik A, Zaidman A, Germán DM, Gonzalez-Barahona J (2020) How bugs are born: a model to identify how bugs are introduced in software components. Empir Softw Eng 25(2):1294–1340 10.1007/s10664-019-09781-y

[CR38] Rodríguez-Pérez G, Nagappan M, Robles G (2022) Watch out for extrinsic bugs! a case study of their impact in just-in-time bug prediction models on the OpenStack project. IEEE Trans Softw Eng 48(4):1400–1416. 10.1109/TSE.2020.3021380 10.1109/TSE.2020.3021380

[CR39] Rodríguez-Pérez G, Zaidman A, Serebrenik A, Robles G, González-Barahona JM (2018) What if a bug has a different origin? making sense of bugs without an explicit bug introducing change. In Proceedings of the 12th ACM/IEEE international symposium on empirical software engineering and measurement, pp 1–4

[CR40] Rosa G, Pascarella L, Scalabrino S, Tufano R, Bavota G, Lanza M, Oliveto R (2021) Evaluating szz implementations through a developer-informed oracle. In 2021 IEEE/ACM 43rd international conference on software engineering (ICSE), pp 436–447. IEEE

[CR41] Śliwerski J, Zimmermann T, Zeller A (2005) When do changes induce fixes? ACM SIGSOFT Softw Eng Notes 30(4):1–5 10.1145/1082983.1083147

[CR42] Spadini D, Aniche M, Bacchelli A (2018) Pydriller: Python framework for mining software repositories. In Proceedings of the 2018 26th ACM Joint Meeting on European Software Engineering Conference and Symposium on the Foundations of Software Engineering, pp 908–911

[CR43] Triola MF, Goodman WM, Law R, Labute G (2006) Elementary statistics. Pearson/Addison-Wesley Reading

[CR44] Turhan B, Menzies T, Bener AB, Di Stefano J (2009) On the relative value of cross-company and within-company data for defect prediction. Empir Softw Eng 14(5):540–578 10.1007/s10664-008-9103-7

[CR45] Wan Z, Xia X, Hassan AE, Lo D, Yin J, Yang Xiaohu (2020) Perceptions, expectations, and challenges in defect prediction. IEEE Trans Softw Eng 46(11):1241–1266. 10.1109/TSE.2018.2877678 10.1109/TSE.2018.2877678

[CR46] Wang X, Dang Y, Zhang L, Zhang D, Lan E, Mei H (2012) Can i clone this piece of code here? In Proceedings of the 27th IEEE/ACM international conference on automated software engineering, pp 170–179

[CR47] Wang Q, Parnin C, Orso A (2015) Evaluating the usefulness of IR-based fault localization techniques. In Proceedings of the 2015 international symposium on software testing and analysis, pp 1–11

[CR48] Wehaibi S, Shihab E, Guerrouj L (2016) Examining the impact of self-admitted technical debt on software quality. In 2016 IEEE 23Rd international conference on software analysis, evolution, and reengineering (SANER), vol 1, pp 179–188. IEEE

[CR49] White J, Hays S, Fu Q, Spencer-Smith J, Schmidt DC (2023) Chatgpt prompt patterns for improving code quality, refactoring, requirements elicitation, and software design. arXiv:2303.07839

[CR50] Wu R, Zhang H, Kim S, Cheung SC (2011) Relink: Recovering links between bugs and changes. ESEC, FSE ’11, New York, NY, USA, pp 15–25, Association for Computing Machinery. ISBN 9781450304436

[CR51] Young J-G, Casari A, McLaughlin K, Trujillo MZ, Hébert-Dufresne L, Bagrow JP (2021) Which contributions count? Analysis of attribution in open source. In 2021 IEEE/ACM 18th international conference on mining software repositories (MSR), pp 242–253. IEEE

